# Neurofibromatosis with vitiligo: an uncommon association rather than coexistence?^☆^^☆☆^

**DOI:** 10.1016/j.abd.2019.09.003

**Published:** 2019-09-30

**Authors:** Sidharth Tandon, Ajeet Singh, Pooja Arora, Ram Krishan Gautam

**Affiliations:** Dermatology Department, PGIMER& Dr. Ram Manohar Lohia Hospital, New Delhi, India

Dear Editor,

Type 1 neurofibromatosis (NF1) is an autosomal dominant multisystem disease caused by a mutation in the neurofibromin 1 gene which affects tissues derived from the neural crest.^1^ Clinically, it is characterized by a spectrum of defects comprising of neural tumors, *cafe-au-lait* spots, intertriginous freckling, and skeletal defects. Generalized vitiligo has rarely been reported with neurofibromatosis. Here, we present two cases of NF1 associated with vitiligo and showing the halo phenomenon in neurofibromas.

A 28-year-old male patient who was a known case of NF1 presented with multiple depigmented patches on the skin, which had started developing in the last four years. His family history revealed that his mother also had neurofibromatosis, with no history of any depigmented lesions. Dermatological examination revealed multiple well-demarcated, light-brown macules of size varying from 5 to 50 mm present over the trunk, back, and upper limbs. Numerous sessile as well as pedunculated dome-shaped papulonodular lesions of varying sizes, suggestive of neurofibromas, were present over the face, trunk, back, and upper limbs. Some of these lesions were encircled by a depigmented halo. Sharply defined depigmented patches, consistent with a diagnosis of vitiligo and varying between 2 and 7 cm in size, were present over back, elbows, and dorsum of hands and feet (Fig. 1). Bilateral axillary freckling was also seen. Slit lamp examination of iris showed the presence of Lisch nodules. Dermoscopy of the vitiligo lesion showed reduced pigmentary network as compared to normal skin (Fig. 2).

**Figure 1:**
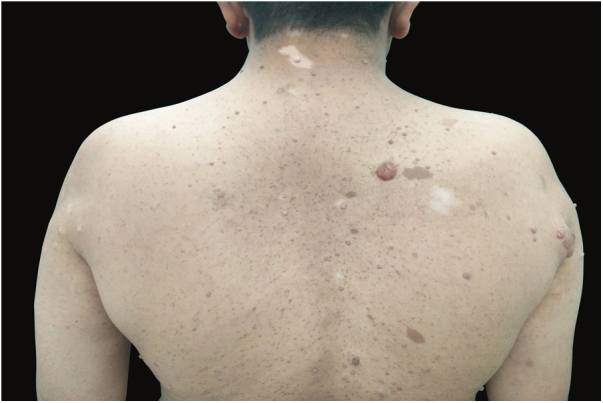
*Café-au-lait* macules, neurofibromas with perilesional halo, and vitiligo patches over the back.

**Figure 2:**
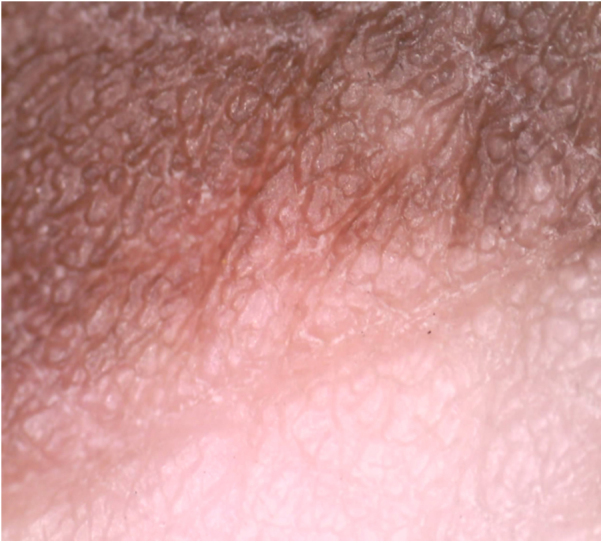
Border of a lesion on dermoscopy, showing reduced pigmentary network in the lesion compared to normal skin.

The second case was a 35-year-old male patient; a known case of neurofibromatosis presented with depigmented patches involving the normal skin and the skin surrounding the neurofibromas over the previous year. There was a family history of neurofibromatosis in his father and brother. On cutaneous examination, multiple dome-shaped, skin colored papulonodular lesions of variable sizes and soft in consistency were present over the face, trunk, back, and extremities. Some nodules had depigmented halo around them. Multiple well-demarcated, light brown colored patches, 5–20 mm in size, were present over back, chest and upper extremities. Sharply demarcated patches of depigmentation suggestive of vitiligo, along with freckles, were present in both the axilla and over the trunk and back. Dermoscopy of the lesion showed reduced pigmentary network.

NF1 is one of the most common autosomal dominant neurocutaneous disorder, with an estimated prevalence of around 1 in 3500 individuals. It has a highly variable clinical expression and approximately 30–50% of all patients lack a family history of the disease, representing *de novo* mutations of the NF1 gene.

Friedrich Von Recklinghausen was the first to describe the classical features of the disease and pointed out that the origin of the skin tumor was from peripheral nerves. *Café-au-lait* macules (CALMs) which are the first sign of NF1, signify the collection of heavily pigmented melanocytes originating from the neural crest in the epidermis. Ninety-five percent of patients of NF1 have CALMs by the time they reach the adulthood. Regarding the pigmentary changes, it was suggested that since the pigment producing melanocyte originates in the neural crest, the presence of pigmentary lesions due to changes in melanocyte cell growth and differentiation can be expected. Cell culture studies have shown that the NF1 gene defect affects melanogenesis in the epidermal melanocytes of NF1 patients, resulting in the various hyperpigmentary changes seen in NF1.^2^

The halo phenomenon signifies the sudden development of a depigmented halo around a congenital nevus, Spitz nevus, blue nevus, neurofibromas, and malignant melanomas. Shin et al.^3^ described a case of naevus combined with vitiliginous changes and speculated that the immunological process involved in vitiligo simultaneously affected the underlying dermal naevus cells, causing their degeneration. Gach et al.^4^ pointed out that clinical and histological similarity between the congenital melanocytic nevi and neurofibromas was because of the fact that both melanocytes and Schwann cells originate from the neural crest (Table 1). The levels of chronically activated CD8^+^ T-cells are increased in NF1 patients, which provides further evidence that the halo phenomenon in neurofibromas in the presence of generalized vitiligo develops as an immunological response.^5^

**Table 1:** Previously reported cases of type 1 neurofibromatosis (NF1) associated with vitiligo.

Year	Author	Type of NF	Type of vitiligo	Associated halo phenomenon	Other associated features
1992	Singh et al.	NF1	Generalized	Absent	
2006	Oiso et al.	NF1	Generalized	Present	
2006	Bukhari et al.	NF1	Generalized	Absent	Left occipital bone defect
2006	Yalccin et al.	NF1	Generalized	Absent	Hashimoto's thyroiditis, Noonan syndrome
2008	Nanda	NF1	Generalized	Absent	
2016	Duman et al.	NF1	Generalized	Absent	Inferior chorioretinal coloboma and optic disc pseudo-doubling in the right eye
2016	Reinehr et al.	NF1	Acral	Absent	
2018	Present	NF1	Generalized	Present	

A thorough review of the literature yielded the following cases of neurofibromatosis associated with vitiligo as described in table 1. The sudden appearance of the halo phenomenon in patients of neurofibromatosis should not be treated as a mere co-existence, but as a marker of a much more sinister association between neurofibromatosis and vitiligo. We recommend that further studies should be carried out to provide a greater insight into this complex association.

## Financial support

None.

## Author's contribution

Sidharth Tandon and Pooja Arora: Approval of the final version of the manuscript; elaboration and writing of the manuscript; critical review of the literature; critical review of the manuscript.

Ajeet Singh: Approval of the final version of the manuscript; conception and planning of the study; elaboration and writing of the manuscript; obtaining, analyzing and interpreting the data; effective participation in research orientation; critical review of the literature; critical review of the manuscript.

Ram Krishan Gautam: Critical review of the literature; critical review of the manuscript.

## Conflicts of interest

The authors declare no conflicts of interest.
